# *In Vitro* and *In Vivo* Evaluation of Chitosan/HPMC/Insulin Hydrogel for Wound Healing Applications

**DOI:** 10.3390/bioengineering11020168

**Published:** 2024-02-09

**Authors:** Flávia Cristina Zanchetta, Pieter De Wever, Joseane Morari, Rita Caiado Gaspar, Thaís Paulino do Prado, Tess De Maeseneer, Ruth Cardinaels, Eliana Pereira Araújo, Maria Helena Melo Lima, Pedro Fardim

**Affiliations:** 1 Faculty of Nursing, University of Campinas, Campinas 13083-887, Brazil; flaviac.zanchetta@gmail.com (F.C.Z.); thaispaulino.prado@live.com (T.P.d.P.); earaujo@unicamp.br (E.P.A.); mhmelolima@gmail.com (M.H.M.L.); 2 Laboratory of Cell Signaling, Obesity and Comorbidities Research Center, University of Campinas, Campinas 13083-887, Brazil; morarij@gmail.com; 3 Department of Chemical Engineering, University of Leuven KU Leuven, 3001 Leuven, Belgium; pieter.dewever@kuleuven.be (P.D.W.); rita.caiadogaspar@kuleuven.be (R.C.G.); tess.demaeseneer@kuleuven.be (T.D.M.); ruth.cardinaels@kuleuven.be (R.C.)

**Keywords:** wound healing, hydrogels, biopolymers, polysaccharides, insulin

## Abstract

Treatment of chronic wounds is challenging, and the development of different formulations based on insulin has shown efficacy due to their ability to regulate oxidative stress and inflammatory reactions. The formulation of insulin with polysaccharides in biohybrid hydrogel systems has the advantage of synergistically combining the bioactivity of the protein with the biocompatibility and hydrogel properties of polysaccharides. In this study, a hydrogel formulation containing insulin, chitosan, and hydroxypropyl methyl cellulose (Chi/HPMC/Ins) was prepared and characterized by FTIR, thermogravimetric, and gel point analyses. The in vitro cell viability and cell migration potential of the Chi/HPMC/Ins hydrogel were evaluated in human keratinocyte cells (HaCat) by MTT and wound scratch assay. The hydrogel was applied to excisional full-thickness wounds in diabetic mice for twenty days for in vivo studies. Cell viability studies indicated no cytotoxicity of the Chi/HPMC/Ins hydrogel. Moreover, the Chi/HPMC/Ins hydrogel promoted faster gap closure in the scratch assay. In vivo, the wounds treated with the Chi/HPMC/Ins hydrogel resulted in faster wound closure, formation of a more organized granulation tissue, and hair follicle regeneration. These results suggest that Chi/HPMC/Ins hydrogels might promote wound healing in vitro and in vivo and could be a new potential dressing for wound healing.

## 1. Introduction

The skin’s role in sensation and protection of the body from environmental hazards is vital. This barrier is designed to prevent microorganism invasion, radiological/chemical damage, and dehydration. Skin injuries result in a complex biologic process that integrates inflammation, mitosis, angiogenesis, extracellular matrix synthesis, and remodeling [1]. A medical condition is usually caused by the failure of one or more of these cellular processes, such as diabetes and vascular disease [2]. However, new drugs or formulations with wound healing potential have been researched as result of the need for less costly and more efficient treatments.

Insulin is a peptide anabolic hormone and growth factor synthesized and secreted by the beta cells of the pancreas that elicits metabolic effects throughout the body [3]. It regulates glucose and lipid metabolism, protein synthesis, mitochondrial biogenesis, cell growth, proliferation, differentiation, and migration in many tissues [4]. In skin, epidermal and dermal human cell growth is dependent on insulin [5].

Insulin’s effects on wound healing started to be observed in the 1930s, when it was noticed that systemic insulin treatment could reduce surgical site infections in diabetic patients. Although the treatment had side effects such as hypoglycemia and hypokalemia. Later, in the 1960s, it was noticed that topical insulin use improved pressure ulcer healing without affecting blood glucose levels [4,5].

In recent decades, the effects of topical insulin on cellular and molecular mechanisms in wound healing have been studied through in vivo and in vitro studies. Among the effects found, it is evident that insulin accelerates re-epithelialization by increasing keratinocyte migration and differentiation. Chen et al. [6] showed that topical insulin could improve healing by regulating the quantity and function of macrophages. These kinds of cells contribute to the secretion of inflammatory mediators responsible for the wound inflammatory phase modulation. Furthermore, insulin activates the insulin receptor (IR, IRS)/phosphoinositide 3-kinase (PI3K)/protein kinase B (AKT) pathways. AKT raises vascular endothelial growth factor (VEGF), which induces the phosphorylation and activation of endothelial nitric oxide synthase (eNOS) in bone marrow, resulting in mobilization of endothelial progenitor cells into the circulation, where they contribute to tissue regeneration. Despite the positive effects that topical insulin has on wound healing, it is an unstable molecule that is easily degradable; for that reason, new formulations have been developed to keep its stability and bioactivity.

One of these possible formulations is its incorporation into hydrogels. Hydrogels have a porous structure, and this characteristic makes them suitable for incorporating many bioactive molecules in order to accelerate wound healing [7,8]. Furthermore, hydrogels have good hydrophilicity, biocompatibility, and a three-dimensional (3D) structure, which resemble those of the skin extracellular matrix (ECM) [7].

Hydrogels can be prepared from natural polymers, synthetic polymers, or a mix of these materials [9,10]. Among natural compounds, chitosan has attracted researchers’ attention due to its properties [11]. This is a hydrophilic polymer [12], and it has amino groups which make it an appropriate compound for the encapsulation and delivery of active ingredients such as insulin [13], magnesium hydroxide [14], LL-37 peptide [15], honey [11], and many others [16,17,18]. Chitosan can act in all stages of wound healing. In the coagulation and hemostasis phase, it can prevent exsanguination. In the inflammatory phase, it regulates the secretion of inflammatory mediators and enhances the function of leukocytes, macrophages, and neutrophils, leading to a microenvironment propitious for healing. In the proliferative phase, this natural polymer stimulates fibroblasts’ proliferation and collagen deposition and promotes angiogenesis, which could contribute to scar prevention in the remodeling phase [19].

Despite these advantages, chitosan has limited stability and low mechanical strength, which could hinder its application for topical gels. To overcome these potential drawbacks, chitosan can be blended with other polysaccharides such as hydroxypropyl methylcellulose (HPMC) [19]. HPMC belongs to the group of cellulose ethers [20], as a semisynthetic nonionic water-soluble polymer [21]. HPMC forms a gelatinous layer, which regulates water transport in the system, contributing to the controlled release of substances from the hydrogel. Additionally, incorporating HPMC into chitosan might be a beneficial approach to enhance the mechanical strength of the hydrogel to provide greater therapeutic efficiency [22].

In this work, we have designed and prepared a formulation containing chitosan, HPMC, and insulin (Chi/HPMC/Ins) for wound healing applications. The goal was to obtain stable gels with advanced wound healing properties.

## 2. Materials and Methods

### 2.1. Materials

Chitosan (Pharmaceutical grade, molecular weight 190–310 kDa, deacetylation degree: ≥75%) was purchased from Sigma-Aldrich Inc. (St. Louis, MO, USA). 3-(4, 5-dimethylthiazol-2-yl)-2,5 diphenyl tetrazolium bromide (MTT); Dulbecco’s modified Eagle’s medium–nutrient mixture F-12 (DMEM/F12) and fetal bovine serum (FBS) were purchased from Gibco, BRL (Eggenstein, Germany). Streptozotocin was obtained from Sigma-Aldrich Inc. (St. Louis, MO, USA). Hydroxypropyl methyl cellulose (HPMC), with an average molecular weight of 86 kDa, was obtained from Sigma-Aldrich Inc. (St. Louis, MO, USA). Regular Insulin (Novolin^®^) was made by Novo Nordisk A/S Bagsvaerd.

### 2.2. Hydrogel Preparation

To prepare Chi/HPMC/Ins hydrogels, chitosan and HPMC were separately dispersed in deionized water and the compounds were gently stirred at 60 °C for 30 min. The chitosan and HPMC solutions were mixed with a ratio of 2:1 *v*/*v*, and then 3 mL of glycerol was added to 18 mL of Chi/HPMC solution. The glycerol was used as a vehicle because it is cosmetically acceptable, has a high viscosity index, is soluble with insulin [23] and can improve the hydrogel’s mechanical properties [24]. The prepared solution was mixed for 1 h at 60 °C. After the mixture had cooled, the human regular insulin was added to the Chi/HPMC solution, and then it was mixed for 1 h more at room temperature. The final insulin concentration was 2 U/g. This mixture was spread on aluminum foil, frozen in liquid nitrogen and placed in a Lyoquest −85 freeze dryer. The lyophilized sample was characterized through FTIR, TGA, and SEM.

### 2.3. Characterization

FTIR spectra were collected using a Bruker Alpha FTIR-ATR, using four scans on the dried gel. Dried gels were also characterized by thermogravimetric analysis utilizing a TGA Q500 (TA Instruments) under a nitrogen atmosphere with a heating rate of 10 °C/min towards a temperature of 550 °C. The surface and cross-section of the film were coated with a layer of gold/palladium for 40 s at 30 mA and imaged using a JEOL JSM-6010 scanning electron microscope.

The gel point of the Chi/HPMC/Ins solutions was determined using a stress-controlled Anton Paar MCR702 rheometer. The home-made plate–plate geometry used consisted of a roughened aluminum bottom plate connected to a Peltier plate, as well as a 25 mm diameter roughened aluminum upper plate. The plates were roughened to avoid wall slip. To closely mimic the application of the gel to a wound, it was explicitly decided not to perform any evaporation control. A time sweep at a constant strain of 0.1% and a frequency of 10 rad/s was performed at a temperature of 37 °C to determine the gel point. The gel point is defined as the time it takes for the storage modulus (G′) to reach and exceed the value of the loss modulus (G″).

### 2.4. Cell Viability Studies

Cell viability studies were assessed using a 3-(4,5-dimethyl-2-thiazolyl)-2,5-diphenyl-2H-tetrazoline bromide (MTT) assay on the HaCat cell line. Briefly, cells were seeded in 12-well plates at 1 × 10^4^ cells/well and maintained at 37 °C with 5% CO_2_ in DMEM containing 10% bovine fetal serum (BFS). Then, the medium was removed and replaced with a fresh one containing Chi/HPMC/Ins hydrogel or Chi/HPMC hydrogel. The DMEM medium was used as a negative control. After 24 h, 48 h, and 72 h after cell seeding, the culture medium was removed from the 12-well plate and 150 μL of MTT (0.5 mg/mL) was added to each well. Afterwards, the cells were incubated at 37 °C for 3–4 h in a dark place; then, the solution was removed, and 0.1 mL DMSO was added to each well. The relative absorbance at 490 nm was measured using a Varioskan Flash microplate reader (Thermo Scientific, Waltham, MA, USA). The cell viability% was calculated using the following equation:

$$Cell\;viability\;(\%)=\frac{A\;sample}{A\;control}\;\times 100$$

where A sample and A control indicate the absorbance of the sample and control wells, respectively. The experiments were performed in triplicate.

### 2.5. Wound Scratch

HaCaT cells were seeded in a 12-well plate at a cell density of 3 × 10^5^ cells/mL until a 90% confluent cell monolayer was obtained. The cell monolayer was scratched in a straight line with a p200 micropipette tip. The debris was removed by washing cells with phosphate-buffered solution, which was replaced with 2 mL of culture medium containing Chi/HPMC hydrogel or Chi/HPCM/Ins hydrogel. In the negative control group, cells were treated with DMEM-1% FBS. Photographs of the wounded area were taken at 24 h and 48 h to investigate and analyze the scratch wound. The percentage of wound closure was calculated using ImageJ 1.49v software (National Institutes of Health, Bethesda, MD, USA) and expressed as reported in the following equation [14]:

$$Wound\;closure\;(\%)=\frac{At-A0}{At}\;\times \;100$$

where A0 is the area of the wound measured immediately after scratching, and At is the area of the wound measured after 24 h or 48 h. All assays were carried out in triplicate.

### 2.6. Wound Healing In Vivo

All animal experiments were approved by the Ethical Committee for Animal Research (approval number 5695-1/2021) at the State University of Campinas (Brazil). Male C57BL/6 mice were purchased from the Breeding Centre of the State University of Campinas (Brazil). Diabetes mellitus (DM) was induced in 6-week-old C57BL/6 mice by intraperitoneal streptozotocin (STZ) injections (50 mg/kg) for 5 consecutive days. The animals were fasted from food 4 h prior to each administration. After three weeks, the caudal vein blood was collected, and the blood glucose was measured with a glucometer (Accu-Check). The criterion for developing DM was defined as a blood glucose level ≥250 mg/dL. The diabetic animals were randomly divided into three treatment groups: the saline group (SAL), treated with physiological saline solution; Chi/HPMC, treated with the hydrogel; and Chi/HPMC/Ins, treated with the hydrogel containing insulin. Then, under general anesthesia, 100 mg/kg ketamine and 10 mg/kg xylazine were administered intraperitoneally. After that, the dorsal region of the mice was shaven and depilated to expose the dorsal skin. A plastic mold of 1 cm^2^ was used to create an excisional full-thickness dorsal skin wound. The wounds were treated daily according to the respective group. Photographs of the wound site were taken at 0, 3, 7, 10, 14, 17 and 20 days after surgery. Wound contraction was measured using ImageJ 1.49v software (National Institutes of Health, Bethesda, MD, USA). The blood glucose and the body weight were also monitored at these times. On days 7, 14, and 20 after surgery, the mice were euthanized. The wounded skin tissues were collected and fixed in 4% paraformaldehyde, dehydrated using an ethanol gradient, embedded in paraffin wax, and cut into 5 μm sections using a microtome. Thus, sections were stained with hematoxilin and eosin (H&E) to observe the morphological structure of the granulation tissue and epidermis.

### 2.7. Statistical Analysis

Results are presented as the mean ± standard deviation (SD) for each experimental group. Statistical comparisons of the data were performed by one-way ANOVA followed by Tukey’s post-test using GraphPad Prism software version 5.0. Differences between groups were considered significant when *p* ≤ 0.05.

## 3. Results

### 3.1. Characterization

**Figure 1:**
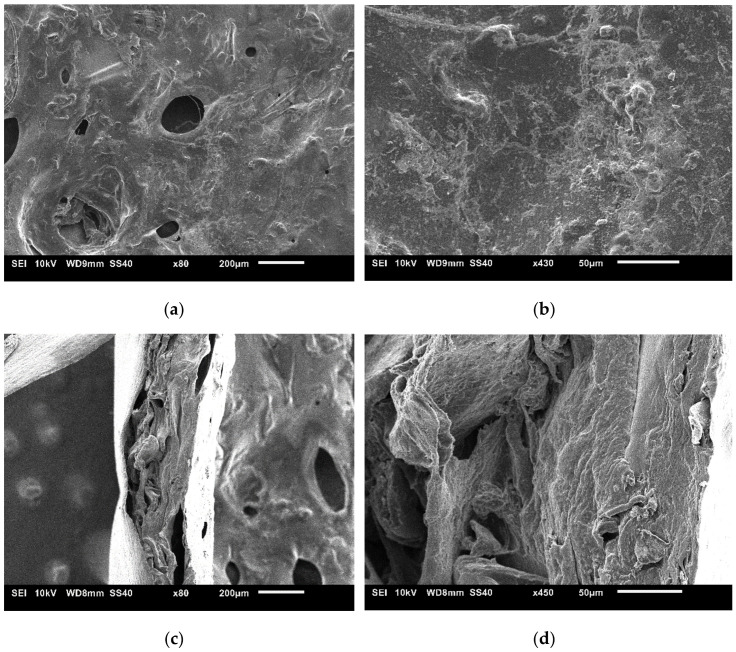
Surface (**a**,**b**) and cross-section (**c**,**d**) of the dried Chi/HPMC/Ins film.

The Chi/HPMC/Ins hydrogel was spread evenly on aluminum foil to create a thin film and freeze-dried to permit chemical characterization and imaging. The sparse distribution of large macropores and the presence of fibrillar structures on the film surface are notable. Furthermore, the globular features suggest that there is heterogeneity in the sample (Figure 1a,b). The cross-section (Figure 1c,d) reveals distinct layers in the sample. From left to right, there is a thin aluminum layer, a core structure with distinct layers, and a denser outer layer. The latter is the surface shown in Figure 1a,b. The porosity in the core is defined by the large cavities existing between layers and microcavities inside the layers. These microcavities are considerably smaller compared to the pores on the surface.

**Figure 2:**
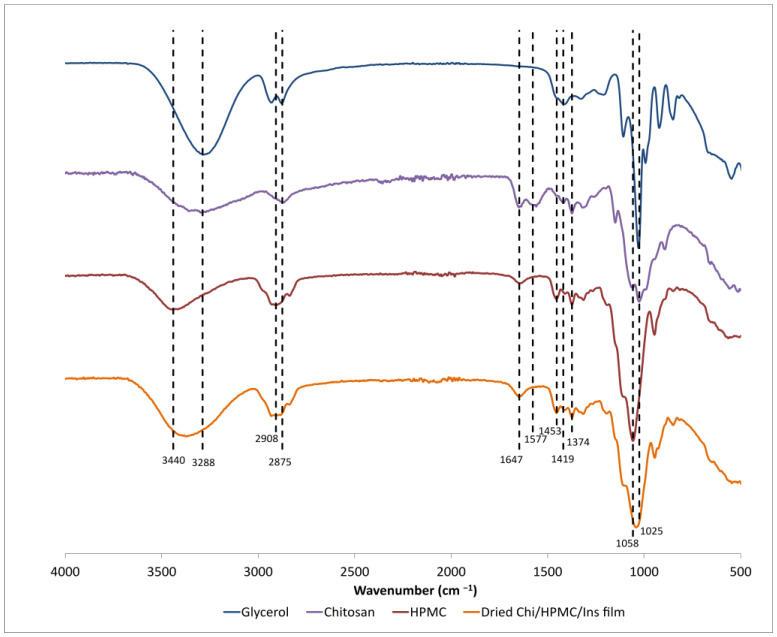
FTIR-ATR spectra of dry chitosan, HPMC, and the dried Chi/HPMC/Ins film.

The ATR-FTIR spectra of chitosan, HPMC, and dried Chi/HPMC/Ins gels are presented in Figure 2. For chitosan, the broad absorption signal spanning 3600 cm^−1^–3100 cm^−1^ indicates vibrations related to O-H and N-H stretching, while the peak found at 2875 cm^−1^ is associated with C-H stretching vibrations [25]. Absorption peaks at 1647 cm^−1^ mark the presence of C=O vibrations of amide II groups, and those at 1577 cm^−1^ indicate the presence of N-H vibrations in amine groups. The peaks at 1419 cm^−1^ and 1374 cm^−1^ originate from C-H vibrations, while both peaks at 1058 cm^−1^ and 1025 cm^−1^ are related to C-O stretching [25,26]. For HPMC, there exists an absorption band spanning 3600 cm^−1^–3100 cm^−1^ which is associated solely with O-H stretching vibrations. The absorption peak at 2908 cm^−1^ marks C-H stretching vibrations in HPMC. The peak at 1453 cm^−1^ is attributed to methylene groups. At 1375 cm^−1^, there is the peak related to O-H bending of the alcohol stretching vibrations, and at 1058 cm^−1^, there is a peak related to C-O stretching vibrations [27,28]. In the Chi/HPMC/Ins film, characteristic peaks of HPMC are clearly observed. In addition, there is broadening of the characteristic peaks at 3365 cm^−1^, 2900 cm^−1^, and 1643 cm^−1^, indicating the presence of chitosan. For insulin, it is expected that amide groups generate absorption bands around 1652 cm^−1^ (amide I) and 1540 cm^−1^ (amide II) [29,30]. For amide I, there is overlap with the signal of chitosan, while presence of an absorption band at 1540 cm^−1^ was not observed. A possible explanation is the low concentration of insulin in comparison with other components of the gel or the location of the insulin in the core of the film, i.e., at depths higher than 1 μm that are not accessible using the FTIR-ATR technique. We hypothesize that the gel structure comprises an interpenetrating network of HPMC and chitosan with the inclusion of insulin.

**Figure A1:**
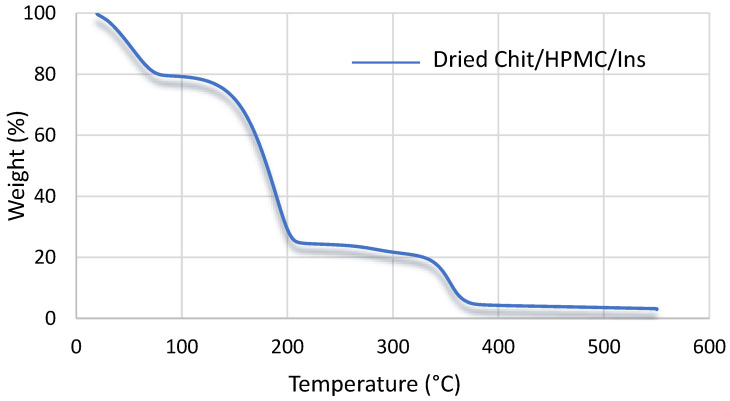
Weight loss as a function of temperature for the dried Chi/HPMC/Ins.

**Figure A2:**
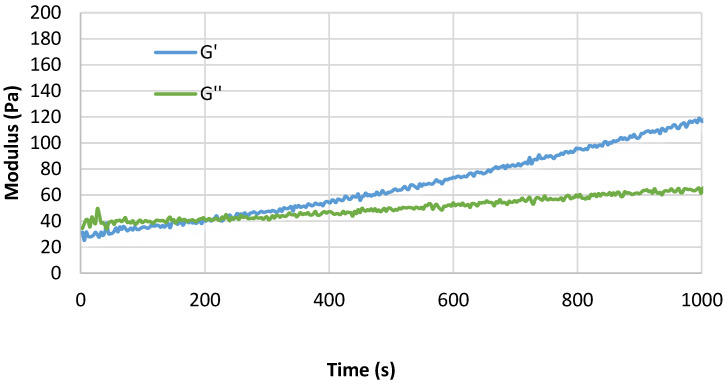
Changes in the storage and loss modulus of Chi/HPMC/Ins solutions, with the gel point at the crossover point of G′ and G″.

When heating the samples up to 100 °C, weight losses due to water are observed, as seen in Figure A1. The reason for weight loss at the beginning of the temperature increase is the evaporation of adsorbed water on the surface of the sample [31]. There is an initial drop in the TGA graph because of the removal of adsorbed water below 100 °C [31]. Degradation of chitosan and HPMC is expected above 200 °C [32,33]. Glycerol has a boiling point of 290 °C, but the presence of water reduces the boiling point [34]. Therefore, the drop in weight between 110 °C and 210 °C is possibly attributed to the removal of entrapped glycerol containing small quantities of water. The final transition with the onset at 335 °C marks the degradation of the polysaccharides [32,33]. A second decrease marks decomposition of the polysaccharides around 338.5 ± 3.6 °C. The rheological experiment confirms that a gel film forms at 37 °C. According to the graph displaying changes in the loss (G″) and storage moduli (G′) as a function of time, which is shown in Figure A2 (Appendix A), the gel point is 240 ± 155 s.

### 3.2. Chi/HPMC/Ins Hydrogel Enhances the Cellular Viability of Human Keratinocyte HaCaT

**Figure 3:**
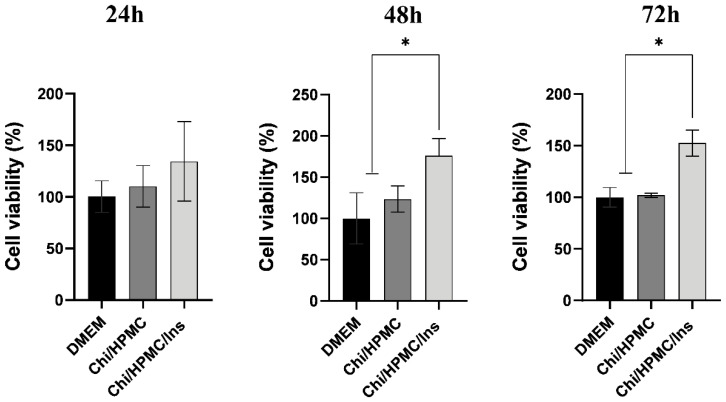
Effect of Chi/HPMC/Ins hydrogel on the cell viability of human keratinocytes (HaCaT). An increase in the cell viability of keratinocytes was produced by the Chi/HPMC/Ins hydrogel after 24 h, 48 h and 72 h of treatment, as evidenced by the MTT assay method. Values are represented as the mean ± SD tested by one-way ANOVA with Tukey’s post hoc test. The experiments were performed in triplicate. * *p* < 0.05.

To investigate the proliferation of HaCat cells on Chit/HPMC and Chi/HPMC/Ins hydrogels after 24 h, 48 h, and 72 h of incubation, the MTT assay was performed. This analysis evidenced that the Chi/HPMC/Ins hydrogel is not cytotoxic to human keratinocytes during any of the incubation periods. The Chi/HPMC/Ins hydrogel was able to increase mitochondrial viability after 24, 48, and 72 h of treatment. The Chi/HPMC/Ins hydrogel stimulated a 31.9% increase in viability of after 24 h (*p* = 0.3867), a 76% increase after 48 h (*p* < 0.0001), and a 112% increase after 72 h, compared to the DMEM control group (*p* < 0.0001) (Figure 3).

### 3.3. Chi/HPMC/Ins Hydrogel Stimulates Cell Proliferation and Migration according to the HaCaT Scratch Assay in Keratinocytes

**Figure 4:**
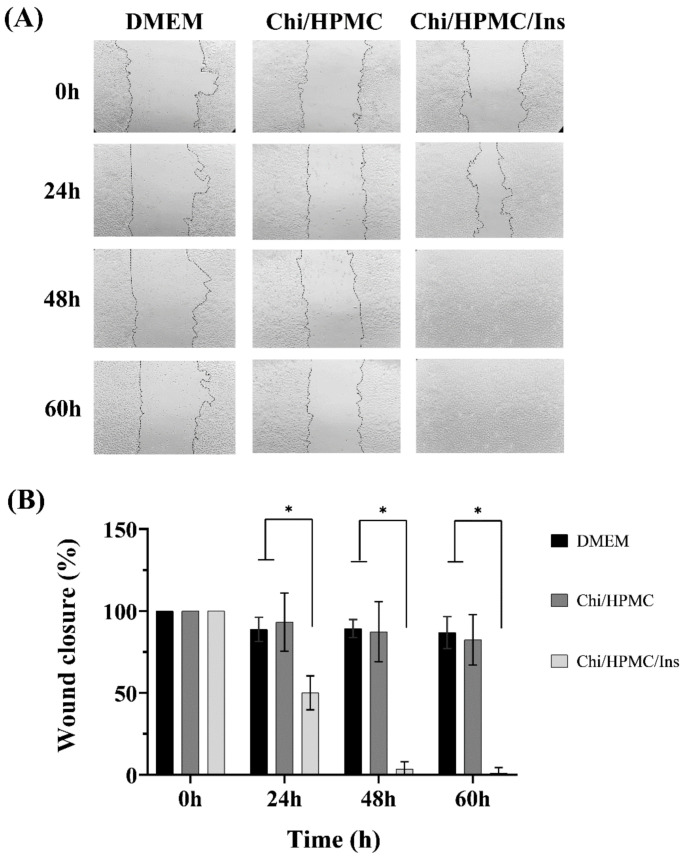
(**A**) Representative images of the HaCat monolayer scratched area with a p200 micropipette tip at 0 h, 24 h, 48 h, and 60 h after different treatments. (**B**) Wound closure expressed via a scratch assay of the cells. The level of significance is denoted as * *p* < 0.0001, as tested by a one-way ANOVA with Tukey’s post hoc test.

To evaluate the effect of Chi/HPMC/Ins on the cell migration and proliferation of HaCaT cells, a time-dependent (0–60 h) wound scratch assay was conducted. To do so, the treatment’s effect on wound healing was analyzed by measuring the areas of wounds immediately after wounding and at four different time points (Figure 4). At 24 h, the wound open areas in the Chi/HPMC/Ins hydrogel, the Chi/HPMC hydrogel, and the DMEM control group were 50%, 93%, and 88%, respectively (*p* < 0.001). At 48 h, the open areas in the Chi/HPMC/Ins hydrogel, the Chit/HPMC hydrogel, and the DMEM were 3%, 87%, and 87%, respectively (*p* < 0.001). At 60 h after treatments, the Chi/HPMC/Ins hydrogel group’s wounded area was completely closed, whereas the wounds in the other groups were still open. The results show that the cells treated with Chi/HPMC/Ins hydrogel migrated significantly compared to those in the Chi/HPMC hydrogel group and the DMEM control group (Figure 4).

### 3.4. The Effects of Chi/HPMC/Ins on Body Weight and Fasting Blood Glucose

**Figure 5:**
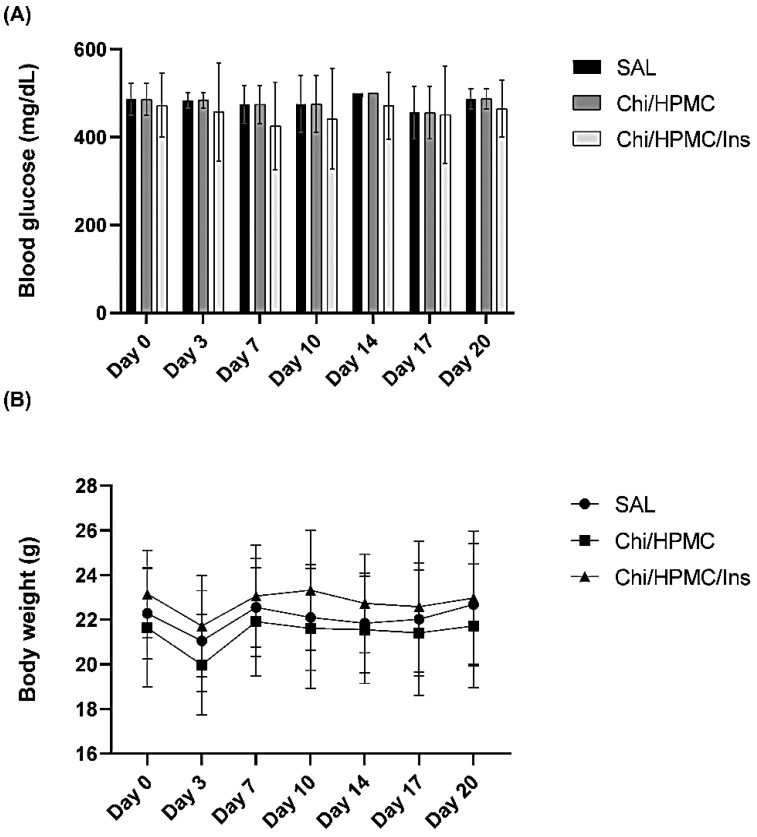
(**A**) Blood glucose levels (mg/dL) on days 0, 3, 7, 10, 14, 17 and 20 after wounding are shown as bar graphs. (**B**) Body weights after wounding at the same time points are depicted as line graphs. The data are expressed as mean ± SD.

Streptozotocin is a medication that destroys the pancreatic islet β-cells, inducing diabetes mellitus. Typical symptoms of diabetes such as polydipsia, polyphagia, and polyuria were observed in the animals after its administration. On day 3 after the wound, the body weight slightly decreased in all experimental groups and returned to initial levels on day 7, which was maintained until the end of the experiment on day 20, as shown in Figure 5. Regarding the blood glucose levels, persistent hyperglycemia was observed in all experimental groups, which confirmed the successful establishment of diabetes in C57BL/6 mice. Furthermore, it shows that the incorporation of insulin into the hydrogel does not affect the blood glucose levels.

### 3.5. Chi/HPMC/Ins Hydrogel Reduces the Area of Full-Thickness Dorsal Skin Wound in Diabetic Mice

**Figure 6:**
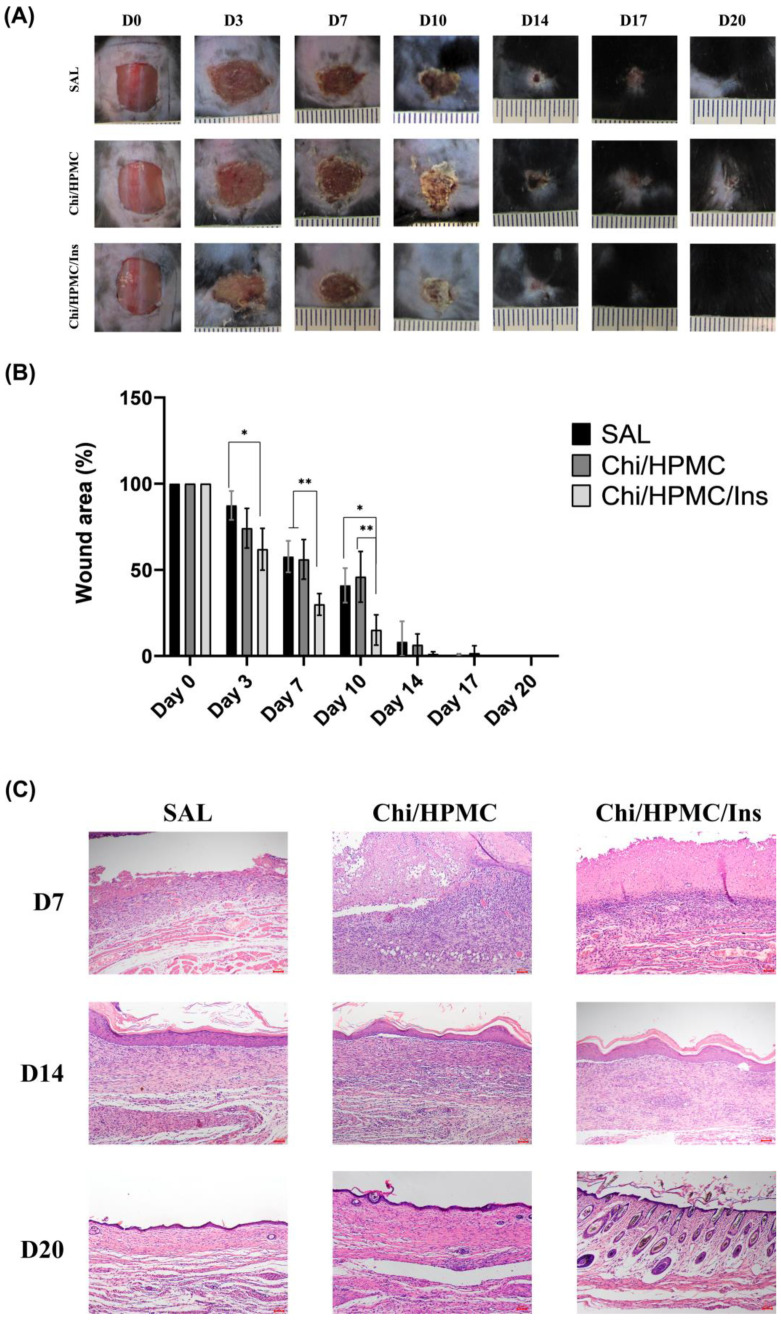
(**A**) Representative pictures of the area contraction of wounds treated with SAL, Chi/HPMC hydrogel, and Chi/HPMC/Ins hydrogel on day 0, 3, 7, 10, 14, 17, and 20 post injury. Scale bar 1 cm. (**B**) Percentage wound contraction. The data are expressed as mean ± SD. The level of significance is denoted * *p* < 0.05 and ** *p* < 0.01, respectively, as tested by a one-way ANOVA with Tukey’s post hoc test. (**C**) Representative images of H&E-stained histological sections on days 7, 14, and 20 after injury. Scale bars: 100 μm.

A full-thickness cutaneous wound in diabetic mice was established to evaluate the efficiency of Chi/HPMC/Ins hydrogels in promoting wound healing. Mice were divided into three groups for in vivo testing: the saline group (negative control group), Chi/HPMC hydrogel group, and Chi/HPMC/Ins hydrogel group. The wound healing was monitored for 20 days and photographs were taken at different time points using images captured with a digital camera (Figure 6A). The Chi/HPMC/Ins hydrogel group presented a tendency towards greater retraction in the wound area on days 3 (*p* < 0.05), 7 (*p* < 0.01), and 10 (*p* < 0.05) compared to the saline group and on days 7 and 10 (*p* < 0.01) and compared to the Chi/HPMC group (Figure 6B).

### 3.6. Histological Assessment

A histological assessment of the wounded tissue in diabetic mice was performed. Tissue sections were stained with H&E to evaluate the wound healing process on days 7, 14, and 20 post wounding. On the seventh day after injury, the Chi/HPMC/Ins hydrogel-treated group exhibited more organized granulation tissue compared to the Chi/HPMC hydrogel and SAL groups. On the 14th day after injury, the tissues had similar characteristics in the three groups. On the 20th day after injury, all groups showed complete re-epithelialization, but the Chi/HPMC/Ins hydrogel group showed hair follicle growth and a smaller scar area (Figure 6C).

## 4. Discussion

It was hypothesized that the Chi/HPMC/Ins hydrogel might act as a wound dressing due to the properties of its components. Several hydrogel wound dressing formulations have been developed due to the advantages of using these materials [13,35,36,37,38].

The three-dimensional structure of hydrogels is comparable to that of the ECM, making them of interest. Hydrogels have high hydrophilicity, which allows them to absorb exudates and contribute to a moist environment. Furthermore, they have high porosity, are biocompatible, have a modifiable degradation rate and a microporous structure network, and have the potential to promote cellular proliferation and migration. All these aspects make them suitable for wound dressings [39,40,41,42,43,44].

The hydrogel developed in this study has chitosan, HPMC, and insulin in its composition. Chitosan is a polysaccharide widely used in the design of wound dressings with hemostatic, anti-bacterial, and fungistatic properties that enhance wound healing [45,46,47].

Hydroxypropyl methylcellulose (HPMC) is a white powder non-ionic cellulose ether that is commonly used in pharmaceutical formulations [48]. The cellulose ether has several important properties such as high tensile strength, and it shows biodegradability and good biocompatibility with natural biopolymers such as chitosan [48]. Insulin is a peptide hormone which exerts functions in different types of body tissue, including the skin. Other studies that investigate topical insulin for wound healing showed it modulates the re-epithelialization process, stimulating cell proliferation and migration of keratinocytes [13,49]. Biodegradable carbohydrate polymers control hydrogel degradation while liberating the hormone in a continued manner [9]. Therefore, the Chi/HPMC/Ins hydrogel seems particularly promising for wound healing.

In this study, the effects of Chi/HPMC/Ins hydrogels were evaluated by in vitro and in vivo analysis. The in vitro results show that Chi/HPMC/Ins hydrogels are not cytotoxic to human keratinocytes. Furthermore, the hydrogel can stimulate cell proliferation and migration of HaCaT with faster wound gap closure in a scratch model. These results corroborate other studies which have found similar results regarding insulin’s effect on HaCat cells [50,51]. In keratinocytes, insulin exerts its effects through PI3K-Akt-Rac1 pathway activation, and this signaling stimulates cell migration. Furthermore, the hydrogels’ structure is like that of skin tissue and the extracellular matrix, which contributes to cellular migration and proliferation, thus leading to faster complete tissue regeneration [39]. The keratinocyte migration and proliferation are essential for successful wound healing [52].

In vivo, the Chi/HPMS/Ins hydrogel contributed to the wound healing process in hyperglycemic mice without affecting blood glucose levels. Other studies have reported the same findings for different types of hydrogels containing insulin, which reinforce our results and the safety of topical insulin use for the treatment of skin lesions [53,54].

Topical insulin has attracted increasing interest as more effective substances are developed for the long-term release of bioactive insulin. The systemic effects of topical insulin can be influenced by its concentration [54]. However, the use of this dose in the study is justifiable because insulin gel enhances wound healing without alterations in glucose level [49].

In hydrogels, insulin is entrapped in the structure, and this allows for prolonged and controlled hormone release into the wound bed [13]. This characteristic could contribute to safety concerning blood glucose levels. Other types of dressings have used insulin in their formulations, and animals’ blood glucose levels have been affected by them [55,56].

Hydrogels have some essential characteristics for wound dressing, such as the ability to keep the wound bed moist, suitable mechanical properties, and biocompatibility. These properties are maintained in the formulation of Chi/HPMC/Ins hydrogels [13].

Additionally, hydrogels can protect the bioactive agents added to them without altering their properties and can control the release of these substances to the wound bed. Furthermore, their structure is like that of skin tissue and the extracellular matrix, which contributes to cellular migration and proliferation, thus leading to faster complete tissue regeneration [18,39,57,58,59].

Hydrogel formulations for wound dressings are well accepted in clinical practice. A meta-analysis examined the healing effectiveness of various types of dressings, such as hydrocolloids, foams, and hydrogels, in diabetic foot ulcers and venous leg ulcers. This study concluded that hydrogel-based dressings were more effective in tissue repair than other types of dressings [60,61].

Insulin use for the treatment of non-healing wounds is widespread in the literature [4,6,9,13,30,49,51,53,54,55,56,62,63,64,65,66,67,68,69,70,71]. Some studies have elucidated the effects of insulin creams, hydrogels, or solutions in the wound-healing process [6,13,54,70]. The results of these studies showed greater inflammatory response, re-epithelialization, and a longer remodeling phase.

This hormone can reduce the wound healing time, modulate the inflammatory phase, and contribute to neogenesis and epithelization. Notably, the repair of all skin structures depends on the combination of cells, signaling molecules, and the extracellular matrix. In addition, in vitro and in vivo studies that seek to understand the specific characteristics of tissue repair and new products are needed to bridge the gap between dressings’ properties and clinical practice.

In this study, the tissue microscopic evaluation showed improved granulation tissue organization after wound treatment with a Chi/HPMC/Ins hydrogel, which indicates greater tissue repair. On the 20th day after the surgery, all experimental groups showed complete epithelization and no inflammation. Full-thickness excisional wounds of diabetic mice have been shown to take 17 days to close. Li et al. analyzed the effects of a pH-responsive hydrogel loaded with insulin on wounds on the feet of diabetic rats [69]. The wounds almost completely healed after 16 days of treatment. The Chi/HPMC/Ins group demonstrated hair follicle regeneration in the wound bed, which could indicate that the dermal papilla has recovered. This structure is crucial for hair follicle regeneration [71]. This finding corroborates that of a similar study which evaluated the effects of a pH-responsive hydrogel loaded with insulin and a keratin-conjugated insulin hydrogel on wound healing [42,69].

Although the application of topical insulin as a wound dressing has been investigated using different types of formulations, such as hydrogels, creams, and fibers [9,49,51,55], there is no consensus regarding the dosage of insulin necessary to promote wound healing. These data can vary in the literature in terms of application frequency and the amount of insulin that each product has. This makes comparisons between studies and the formulations applied difficult [4].

## 5. Conclusions

In vitro assessments showed that Chi/HPMC/Ins hydrogels have very good biocompatibility and increased keratinocyte migration, and they are not cytotoxic for this type of cell. In vivo, the Chi/HPMC/Ins hydrogel group had the highest wound closure rate compared with the Chi/HPMC and SAL groups in hyperglycemic mice. Furthermore, the Chi/HPMC/Ins hydrogel contributed to hair follicle regeneration at the wound site. These results suggest that Chi/HPMC/Ins hydrogels could be a new potential dressing for wound healing. Nevertheless, it is important to consider the practical clinical setting, because in this scenario, wounds have different etiologies, and every patient has intrinsic and extrinsic factors that could interfere in wound healing. Thus, a personalized wound dressing should be designed to address these differences. Nevertheless, it is challenging to translate knowledge from laboratories with controlled environments to clinical practice, wherein different variables may affect wound healing. For that reason, clinical studies should be performed.

## Data Availability

Data are contained within the article.

## References

[1] Childs D.R., Murthy A.S. (2017). Overview of Wound Healing and Management. Surg. Clin. N. Am..

[2] Brocke T., Barr J. (2020). The History of Wound Healing. Surg. Clin. N. Am..

[3] González-Saldivar G., Rodríguez-Gutiérrez R., Ocampo-Candiani J., González-González J.G., Gómez-Flores M. (2017). Skin Manifestations of Insulin Resistance: From a Biochemical Stance to a Clinical Diagnosis and Management. Dermatol. Ther..

[4] Wang J., Xu J. (2020). Effects of Topical Insulin on Wound Healing: A Review of Animal and Human Evidences. Diabetes Metab. Syndr. Obes..

[5] Emanuelli T., Burgeiro A., Carvalho E. (2016). Effects of Insulin on the Skin: Possible Healing Benefits for Diabetic Foot Ulcers. Arch. Dermatol. Res..

[6] Chen X., Liu Y., Zhang X. (2012). Topical Insulin Application Improves Healing by Regulating the Wound Inflammatory Response. Wound Repair Regen..

[7] Liang Y., He J., Guo B. (2021). Functional Hydrogels as Wound Dressing to Enhance Wound Healing. ACS Nano.

[8] Fan F., Saha S., Hanjaya-Putra D. (2021). Biomimetic Hydrogels to Promote Wound Healing. Front. Bioeng. Biotechnol..

[9] Mansoor S., Kondiah P.P.D., Choonara Y.E. (2021). Advanced Hydrogels for the Controlled Delivery of Insulin. Pharmaceutics.

[10] Su J., Li J., Liang J., Zhang K., Li J. (2021). Hydrogel Preparation Methods and Biomaterials for Wound Dressing. Life.

[11] Chopra H., Bibi S., Kumar S., Khan M.S., Kumar P., Singh I. (2022). Preparation and Evaluation of Chitosan/PVA Based Hydrogel Films Loaded with Honey for Wound Healing Application. Gels.

[12] Ahmadi F., Oveisi Z., Samani S.M., Amoozgar Z. (2015). Chitosan Based Hydrogels: Characteristics and Pharmaceutical Applications. Res. Pharm. Sci..

[13] Ostróżka-Cieślik A., Wilczyński S., Dolińska B. (2023). Hydrogel Formulations for Topical Insulin Application: Preparation, Characterization and In Vitro Permeation across the Strat-M^®^ Membrane. Polymers.

[14] Eivazzadeh-Keihan R., Radinekiyan F., Aliabadi H.A.M., Sukhtezari S., Tahmasebi B., Maleki A., Madanchi H. (2021). Chitosan Hydrogel/Silk Fibroin/Mg(OH)_2_ Nanobiocomposite as a Novel Scaffold with Antimicrobial Activity and Improved Mechanical Properties. Sci. Rep..

[15] Yang X., Guo J.-L., Han J., Si R.-J., Liu P.-P., Zhang Z.-R., Wang A.-M., Zhang J. (2020). Chitosan Hydrogel Encapsulated with LL-37 Peptide Promotes Deep Tissue Injury Healing in a Mouse Model. Mil. Med. Res..

[16] Raza Z.A., Khalil S., Ayub A., Banat I.M. (2020). Recent Developments in Chitosan Encapsulation of Various Active Ingredients for Multifunctional Applications. Carbohydr. Res..

[17] Guyot C., Cerruti M., Lerouge S. (2021). Injectable, Strong and Bioadhesive Catechol-Chitosan Hydrogels Physically Crosslinked Using Sodium Bicarbonate. Mater. Sci. Eng. C.

[18] Deng P., Yao L., Chen J., Tang Z., Zhou J. (2022). Chitosan-Based Hydrogels with Injectable, Self-Healing and Antibacterial Properties for Wound Healing. Carbohydr. Polym..

[19] Liu H., Wang C., Li C., Qin Y., Wang Z., Yang F., Li Z., Wang J. (2018). A Functional Chitosan-Based Hydrogel as a Wound Dressing and Drug Delivery System in the Treatment of Wound Healing. RSC Adv..

[20] Deshmukh K., Basheer Ahamed M., Deshmukh R.R., Khadheer Pasha S.K., Bhagat P.R., Chidambaram K. (2017). Biopolymer Composites with High Dielectric Performance: Interface Engineering. Biopolymer Composites in Electronics.

[21] Varshosaz J., Taymouri S., Minaiyan M., Rastegarnasab F., Baradaran A. (2018). Development and In Vitro/In Vivo Evaluation of HPMC/Chitosan Gel Containing Simvastatin Loaded Self-Assembled Nanomicelles as a Potent Wound Healing Agent. Drug Dev. Ind. Pharm..

[22] Chen C.-P., Hsieh C.-M., Tsai T., Yang J.-C., Chen C.-T. (2015). Optimization and Evaluation of a Chitosan/Hydroxypropyl Methylcellulose Hydrogel Containing Toluidine Blue O for Antimicrobial Photodynamic Inactivation. Int. J. Mol. Sci..

[23] Negrini J., Mozos E., Escamilla A., Pérez J., Lucena R., Guerra R., Ginel P.J. (2017). Effects of Topical Insulin on Second-Intention Wound Healing in the Red-Eared Slider Turtle (Trachemys Scripta Elegans)—A Controlled Study. BMC Vet. Res..

[24] Hardman D., George Thuruthel T., Iida F. (2022). Self-Healing Ionic Gelatin/Glycerol Hydrogels for Strain Sensing Applications. NPG Asia Mater..

[25] Fernandes Queiroz M., Melo K., Sabry D., Sassaki G., Rocha H. (2014). Does the Use of Chitosan Contribute to Oxalate Kidney Stone Formation?. Mar. Drugs.

[26] Gradinaru L.M., Barbalata-Mandru M., Enache A.A., Rimbu C.M., Badea G.I., Aflori M. (2023). Chitosan Membranes Containing Plant Extracts: Preparation, Characterization and Antimicrobial Properties. Int. J. Mol. Sci..

[27] Bashir S., Zafar N., Lebaz N., Mahmood A., Elaissari A. (2020). Hydroxypropyl Methylcellulose-Based Hydrogel Copolymeric for Controlled Delivery of Galantamine Hydrobromide in Dementia. Processes.

[28] Akhlaq M., Maryam F., Elaissari A., Ullah H., Adeel M., Hussain A., Ramzan M., Ullah O., Zeeshan Danish M., Iftikhar S. (2018). Pharmacokinetic Evaluation of Quetiapine Fumarate Controlled Release Hybrid Hydrogel: A Healthier Treatment of Schizophrenia. Drug Deliv..

[29] Prusty A.K., Sahu S.K. (2013). Development and Evaluation of Insulin Incorporated Nanoparticles for Oral Administration. ISRN Nanotechnol..

[30] Azevedo J.R., Sizilio R.H., Brito M.B., Costa A.M.B., Serafini M.R., Araújo A.A.S., Santos M.R.V., Lira A.A.M., Nunes R.S. (2011). Physical and Chemical Characterization Insulin-Loaded Chitosan-TPP Nanoparticles. J. Therm. Anal. Calorim..

[31] Wang S.F., Shen L., Tong Y.J., Chen L., Phang I.Y., Lim P.Q., Liu T.X. (2005). Biopolymer Chitosan/Montmorillonite Nanocomposites: Preparation and Characterization. Polym. Degrad. Stab..

[32] Qu X., Wirsén A., Albertsson A.-C. (2000). Effect of Lactic/Glycolic Acid Side Chains on the Thermal Degradation Kinetics of Chitosan Derivatives. Polymer.

[33] Li R., Pan Y., Chen D., Xu X., Yan G., Fan T. (2022). Design, Preparation and In Vitro Evaluation of Core–Shell Fused Deposition Modelling 3D-Printed Verapamil Hydrochloride Pulsatile Tablets. Pharmaceutics.

[34] Takamura K., Fischer H., Morrow N.R. (2012). Physical Properties of Aqueous Glycerol Solutions. J. Pet. Sci. Eng..

[35] Ming Z., Han L., Bao M., Zhu H., Qiang S., Xue S., Liu W. (2021). Living Bacterial Hydrogels for Accelerated Infected Wound Healing. Adv. Sci..

[36] Kushibiki T., Mayumi Y., Nakayama E., Azuma R., Ojima K., Horiguchi A., Ishihara M. (2021). Photocrosslinked Gelatin Hydrogel Improves Wound Healing and Skin Flap Survival by the Sustained Release of Basic Fibroblast Growth Factor. Sci. Rep..

[37] Zhang L., Zhang Y., Ma F., Liu X., Liu Y., Cao Y., Pei R. (2022). A Low-Swelling and Toughened Adhesive Hydrogel with Anti-Microbial and Hemostatic Capacities for Wound Healing. J. Mater. Chem. B.

[38] Chen G., Wang F., Zhang X., Shang Y., Zhao Y. (2023). Living Microecological Hydrogels for Wound Healing. Sci. Adv..

[39] Solanki D., Vinchhi P., Patel M.M. (2023). Design Considerations, Formulation Approaches, and Strategic Advances of Hydrogel Dressings for Chronic Wound Management. ACS Omega.

[40] Kwon J.W., Savitri C., An B., Yang S.W., Park K. (2023). Mesenchymal Stem Cell-Derived Secretomes-Enriched Alginate/Extracellular Matrix Hydrogel Patch Accelerates Skin Wound Healing. Biomater. Res..

[41] Ruffo M., Parisi O.I., Dattilo M., Patitucci F., Malivindi R., Pezzi V., Tzanov T., Puoci F. (2022). Synthesis and Evaluation of Wound Healing Properties of Hydro-Diab Hydrogel Loaded with Green-Synthetized AGNPS: In Vitro and in Ex Vivo Studies. Drug Deliv. Transl. Res..

[42] Li W., Gao F., Kan J., Deng J., Wang B., Hao S. (2019). Synthesis and Fabrication of a Keratin-Conjugated Insulin Hydrogel for the Enhancement of Wound Healing. Colloids Surf. B Biointerfaces.

[43] Feng Z., Su Q., Zhang C., Huang P., Song H., Dong A., Kong D., Wang W. (2020). Bioinspired Nanofibrous Glycopeptide Hydrogel Dressing for Accelerating Wound Healing: A Cytokine-Free, M2-Type Macrophage Polarization Approach. Adv. Funct. Mater..

[44] Qu J., Zhao X., Liang Y., Zhang T., Ma P.X., Guo B. (2018). Antibacterial Adhesive Injectable Hydrogels with Rapid Self-Healing, Extensibility and Compressibility as Wound Dressing for Joints Skin Wound Healing. Biomaterials.

[45] Laurano R., Boffito M., Ciardelli G., Chiono V. (2022). Wound Dressing Products: A Translational Investigation from the Bench to the Market. Eng. Regen..

[46] Keast D.H., Janmohammad A. (2021). The Hemostatic and Wound Healing Effect of Chitosan Following Debridement of Chronic Ulcers. Wounds.

[47] Li Z., Yang F., Yang R. (2015). Synthesis and Characterization of Chitosan Derivatives with Dual-Antibacterial Functional Groups. Int. J. Biol. Macromol..

[48] Pitpisutkul V., Prachayawarakorn J. (2022). Hydroxypropyl Methylcellulose/Carboxymethyl Starch/Zinc Oxide Porous Nanocomposite Films for Wound Dressing Application. Carbohydr. Polym..

[49] Apolinário P.P., Zanchetta F.C., Breder J.S.C., Adams G., Consonni S.R., Gillis R., Saad M.J.A., Lima M.H.M. (2023). Anti-Inflammatory, Procollagen, and Wound Repair Properties of Topical Insulin Gel. Braz. J. Med. Biol. Res..

[50] Liu Y., Petreaca M., Yao M., Martins-Green M. (2009). Cell and Molecular Mechanisms of Keratinocyte Function Stimulated by Insulin during Wound Healing. BMC Cell Biol..

[51] Aijaz A., Faulknor R., Berthiaume F., Olabisi R.M. (2015). Hydrogel Microencapsulated Insulin-Secreting Cells Increase Keratinocyte Migration, Epidermal Thickness, Collagen Fiber Density, and Wound Closure in a Diabetic Mouse Model of Wound Healing. Tissue Eng. Part A.

[52] Fang W.-C., Lan C.-C.E. (2023). The Epidermal Keratinocyte as a Therapeutic Target for Management of Diabetic Wounds. Int. J. Mol. Sci..

[53] Dawoud M.H.S., Yassin G.E., Ghorab D.M., Morsi N.M. (2019). Insulin Mucoadhesive Liposomal Gel for Wound Healing: A Formulation with Sustained Release and Extended Stability Using Quality by Design Approach. AAPS PharmSciTech.

[54] Azevedo F., Pessoa A., Moreira G., Dos Santos M., Liberti E., Araujo E., Carvalho C., Saad M., Lima M.H. (2016). Effect of Topical Insulin on Second-Degree Burns in Diabetic Rats. Biol. Res. Nurs..

[55] Chakraborty T., Gupta S., Nair A., Chauhan S., Saini V. (2021). Wound Healing Potential of Insulin-Loaded Nanoemulsion with Aloe Vera Gel in Diabetic Rats. J. Drug Deliv. Sci. Technol..

[56] Zhu J., Jiang G., Hong W., Zhang Y., Xu B., Song G., Liu T., Hong C., Ruan L. (2020). Rapid Gelation of Oxidized Hyaluronic Acid and Succinyl Chitosan for Integration with Insulin-Loaded Micelles and Epidermal Growth Factor on Diabetic Wound Healing. Mater. Sci. Eng. C.

[57] Wang H., Huang R., Bai L., Cai Y., Lei M., Bao C., Lin S., Ji S., Liu C., Qu X. (2023). Extracellular Matrix-Mimetic Immunomodulatory Hydrogel for Accelerating Wound Healing. Adv. Healthc. Mater..

[58] Lungu R., Paun M.-A., Peptanariu D., Ailincai D., Marin L., Nichita M.-V., Paun V.-A., Paun V.-P. (2022). Biocompatible Chitosan-Based Hydrogels for Bioabsorbable Wound Dressings. Gels.

[59] Bradshaw M., Ho D., Fear M.W., Gelain F., Wood F.M., Iyer K.S. (2014). Designer Self-Assembling Hydrogel Scaffolds Can Impact Skin Cell Proliferation and Migration. Sci. Rep..

[60] Zhang L., Yin H., Lei X., Lau J.N.Y., Yuan M., Wang X., Zhang F., Zhou F., Qi S., Shu B. (2019). A Systematic Review and Meta-Analysis of Clinical Effectiveness and Safety of Hydrogel Dressings in the Management of Skin Wounds. Front. Bioeng. Biotechnol..

[61] Saco M., Howe N., Nathoo R., Cherpelis B. (2016). Comparing the Efficacies of Alginate, Foam, Hydrocolloid, Hydrofiber, and Hydrogel Dressings in the Management of Diabetic Foot Ulcers and Venous Leg Ulcers: A Systematic Review and Metaanalysis Examining How to Dress for Success. J. Am. Acad. Dermatol..

[62] Walther M., Vestweber P.K., Kühn S., Rieger U., Schäfer J., Münch C., Vogel-Kindgen S., Planz V., Windbergs M. (2023). Bioactive Insulin-Loaded Electrospun Wound Dressings for Localized Drug Delivery and Stimulation of Protein Expression Associated with Wound Healing. Mol. Pharm..

[63] Oryan A., Alemzadeh E. (2017). Effects of Insulin on Wound Healing: A Review of Animal and Human Evidences. Life Sci..

[64] Liu Y., Dhall S., Castro A., Chan A., Alamat R., Martins-Green M. (2018). Insulin Regulates Multiple Signaling Pathways Leading to Monocyte/Macrophage Chemotaxis into the Wound Tissue. Biol. Open.

[65] Besson J.C.F., Hernandes L., de Campos J.M., Morikawa K.A., Bersani-Amado C.A., Matioli G. (2017). Insulin Complexed with Cyclodextrins Stimulates Epithelialization and Neovascularization of Skin Wound Healing in Rats. Injury.

[66] Li X., Liu Y., Zhang J., You R., Qu J., Li M. (2017). Functionalized Silk Fibroin Dressing with Topical Bioactive Insulin Release for Accelerated Chronic Wound Healing. Mater. Sci. Eng. C.

[67] Yang P., Wang X., Wang D., Shi Y., Zhang M., Yu T., Liu D., Gao M., Zhang X., Liu Y. (2020). Topical Insulin Application Accelerates Diabetic Wound Healing by Promoting Anti-Inflammatory Macrophage Polarization. J. Cell Sci..

[68] Greenway S.E., Filler L.E., Greenway F.L. (1999). Topical Insulin in Wound Healing: A Randomised, Double-Blind, Placebo-Controlled Trial. J. Wound Care.

[69] Li Z., Zhao Y., Liu H., Ren M., Wang Z., Wang X., Liu H., Feng Y., Lin Q., Wang C. (2021). PH-Responsive Hydrogel Loaded with Insulin as a Bioactive Dressing for Enhancing Diabetic Wound Healing. Mater. Des..

[70] Li C., Yu T., Liu Y., Chen X., Zhang X. (2015). Topical Application of Insulin Accelerates Vessel Maturation of Wounds by Regulating Angiopoietin-1 in Diabetic Mice. Int. J. Low. Extrem. Wounds.

[71] Lima M.H.M., Caricilli A.M., de Abreu L.L., Araújo E.P., Pelegrinelli F.F., Thirone A.C.P., Tsukumo D.M., Pessoa A.F.M., dos Santos M.F., de Moraes M.A. (2012). Topical Insulin Accelerates Wound Healing in Diabetes by Enhancing the AKT and ERK Pathways: A Double-Blind Placebo-Controlled Clinical Trial. PLoS ONE.

